# The Early Infancy of a Parent and Baby Mental Health Unit: A Reflection

**DOI:** 10.1007/s10995-024-04027-w

**Published:** 2024-11-28

**Authors:** Sophie Isobel, Alison Green, Sylvia Lim-Gibson

**Affiliations:** 1 Naamuru Parent and Baby Unit, Royal Prince Alfred Hospital, Sydney Local Health District, Building 23, Cnr Susan & Grose St, Camperdown, NSW 2050 Australia; 2 Faculty of Medicine and Health, University of Sydney, Camperdown, Australia

**Keywords:** Mother baby unit, Parent baby unit, Mental health unit, Service design, Attachment

## Abstract

**Purpose:**

This article describes the experience of establishing a new parent and baby mental health unit, including challenges in the first year of operation. The article aims to narrate the experience for the purpose of informing other new mental health services and contributing to service development knowledge.

**Description:**

The analogy of the early infancy period is used to highlight the parallel process of adjustment, confidence and identity formation occurring as part of the unit development and by the parents who are admitted.

**Assessment:**

Key challenges are presented as *“We had a baby and moved house at the same time”*,* “We had a baby with someone we just met”*,* “We had ghosts in our communal nursery”* and *“We were juggling the baby and the bathwater”.*

**Conclusion:**

The establishment of a new unit provides an opportunity to reflect on the complexity of building workforce, service and clinical capacity within the constraints of public health services. The challenges along the way have helped to build empathy for the experiences of the admitted parents who equally find that things have not gone how they have planned, while finding emerging opportunities for growth, resilience and change.

## Significance

**What is Already Known:**

Mother Baby Units are globally established as the standard of care for providing mental health care to parents experiencing perinatal mental health disorders. They are known to improve parents health, confidence and attachment while providing rewarding places to work for multidisciplinary clinicians.

**What this Article Adds:**

This article reflects on the challenges identified in the establishment of speciality units requiring relational care within existing paradigms of mental health service provision. The articles raises awareness of the complexity of inpatient perinatal care and advocates for systemic awareness of the specialist knowledge, skills, staffing and resources required to provide attachment-focused acute inpatient mental health care to parents and infants.

## Purpose

Perinatal mental illness impacts parents, infants and their families. The early postnatal period is considered a period of high risk for new and recurrent episodes of mental illness, with a small percentage of parents requiring admission to a mental health unit (Howard & Khalifeh, 2020). Onset of perinatal disorders can occur rapidly and deterioration can occur quickly (Trevillion et al., 2019). Perinatal mental illness is associated with adverse outcomes for parents and infants including impaired bonding, loss of parenting confidence, and delayed developmental outcomes (Stein et al., 2014). Across the world, Mother Baby Units (MBUs) are well-established as the standard of care to provide inpatient treatment for parents experiencing acute perinatal mental illness, while supporting parent-infant attachment. Australian MBUs have existed since the 1980s (Galbally et al., 2019), however, the first publicly funded MBU for the state of New South Wales (NSW) opened in inner Sydney in 2022. This article explores lessons learned in the first 12 months of operation using the analogy of early infancy.

## Description

Whilst MBUs are well-established globally, there is no accepted definition of how an MBU should function (Connellan et al., 2017), creating a challenge for adult mental health services who may not previously had to consider how to provide relational inpatient mental health care.

In the first 12 months of operation, a steady flow of patients were admitted to the MBU with high acuity and complex needs, and discharged with decreased distress, increased attachment and confidence. But as is the case with new parents, appearances can be deceiving and efforts towards outputs manifold; thus, while it is usual to document the outputs of new services as part of evaluation and research, this article documents some of the challenges of the journey so far. This reflective article was developed collaboratively by senior staff on the unit in response to the prompt: ‘what have we learnt?’. It does not include any clinical data and ethics approval was not required. While academic articles usually use lexico-grammatical features that imply impersonal processes (Webb, 1992), this paper positions the authors as central to the learnings and processes described and subsequently, the pronoun ‘we’ is used throughout. ‘We’, herein, refers to the authors of this paper who were all in leadership positions throughout the MBU establishment and use this positionality to represent the observed experiences of the staff of the unit. Themed learnings are named using analogous statements.

**“We had a baby and moved house at the same time”**

A process of co-design led to construction of a purpose-developed unit on the site of a large general hospital, adjacent to an adult mental health unit. As for all aspects of parenthood, no amount of reading, observing and imagining can prepare one for the unknown. For the MBU, it was hard to imagine how the space may be used before it was, with many issues arising only in occupancy by a workforce of new staff, up to 8 acutely unwell parents, 8 support people, and at least 8 infants. If we were to build a similar unit today with knowledge of the everyday usage of the environment, the medication room would be twice as large, the breastmilk fridge a third of its size without its industrial noises and lights, and the number of nursery spaces tripled.

The built environments of inpatient units shapes how patients experience them (Molin et al., 2021) Ulrich et al. (2018) identified that single rooms with private bathrooms, adjustable lighting and sound levels, common areas with moveable furniture and limited noise, windows, artwork featuring nature, access to a garden, exposure to daylight, and a spacious and clean environment can improve patient experiences in mental health units generally. The environment of healthcare delivery can impact upon recovery through fostering normalcy, safety, homeliness, and connection (Olausson et al., 2019; Sui et al., 2023). The MBU incorporated all of these design elements, is well equipped with toys and art, is light and spacious, has home-like bedrooms and a large courtyard with play spaces to promote parental wellbeing and infant development. However, like the experiences that can occur within the cleanest or most glamorous of homes, the pleasant environment can also disguise the risks and complexity of perinatal mental illness and the specialised care provided. There is a constant need to balance messaging about the therapeutic environment, which has been described as ‘resort-like’, with articulation of the seriousness of perinatal mental illness. This balance is also represented environmentally, with design elements such as observation windows and doors without ligature points discretely integrated to enable visibility and safety of parents and infants.

**“We had a baby with someone we just met”**

MBUs are staffed by multi-disciplinary teams with expertise in the treatment of perinatal mental illness, supporting parent-infant attachment, and increasing parent confidence (Howard & Khalifeh, 2020). Interventions include medication, therapy, parenting support and education, infant-care and attachment focused parent-infant support and discharge planning (Branjerdporn et al., 2022), undertaken in compassionate, non-judgmental (Casanova Dias et al., 2022), trauma-informed, recovery-oriented, attachment-informed, person-centred and strengths-based ways (Branjerdporn et al., 2022).

In the MBU, without any similar facilities to attract staff from, the workforce largely had no perinatal experience and were recruited from adult inpatient units. Therefore, while staff were familiar with the mental health system and could ensure care of the primary patient, they didn’t necessarily know the nuances of perinatal interventions and all training had to occur on the job. Adult mental health staff are known to lack knowledge, confidence and skills in working with families (Gregg et al., 2021; Maybery et al., 2016) and while MBUs commonly attract passionate staff, a lack of specialist skills, the demands upon them and understaffing can create barriers to quality care (Griffiths et al., 2019). The MBU is staffed by a new team of mental health nurses, child and family health nurses, psychiatry and allied health including social work, psychology, midwifery, occupational therapy, paediatric, exercise physiology and peer support workers. Providing attachment-focused care to acutely unwell parents and their infants was a steep learning curve, undertaken without existing team trust or processes. Over the first 12 months, while staff surveys showed measurable increases in confidence, knowledge and skills, there were also significant challenges, including high staff turnover. A gap between available training and expected skills has been observed across professional groups in perinatal contexts (Casanova Dias et al., 2022). While training exists, it largely targets adjacent services rather than speciality units. Thus, weekly targeted education and professional development was established on the MBU and a purpose-developed orientation program is in development, yet developing and integrating these takes time.

In our experience, alongside the need for training in attachment, parent-infant observation and intervention, and perinatal mental illness for clinicians working in the MBU, there is also a need for training for other mental health staff in assessing and responding to parents, due to the use of rotational and casual staff, extended hours coverage by non-perinatal clinicians and the majority of referrals coming from adult inpatient units.

**“We had ghosts in our communal nursery”**

Without perinatal expertise, staff brought their own beliefs about parenting to the work. Staff in all settings, construe patients through their own worldviews and life experiences, at times leading to judgement, frustration or helplessness (Blundell et al., 2012). The MBU has been challenged by countertransference, seemingly due to similarities between client and staff demographics and the ‘ghosts’ in our communal nursery. As Fraiberg et al.(1975) observed: *“In every nursery there are ghosts. They are the visitors from the unremembered past of the parents*,* the uninvited guests at the christening”* (p387). In the MBU, the nursery is filled with parents’ ghosts as well as those of the staff. Supporting parents to recognize ghosts in their nurseries is a primary focus of many perinatal interventions (Malone & Dayton, 2015) however to do so, those that exist in the team and are activated by engaging in the attachment systems of admitted parents and infants, also require consideration. A focus on nursery ‘angels’ could assist in identifying the unconscious legacies of feeling loved and safe that buffer against intergenerational or attachment trauma (Narayan et al., 2017). It is intended that staff take on the role of nursery angels, modelling responsive and sensitive care for both parents and infants, but staff’s own unconscious attachment experiences, both good and bad, influence care dynamics.

We soon learnt that secrets held in admitted families could become secrets in the team if alliances and unconscious biases weren’t addressed through regular supervision and reflective practice. This related to staff aligning to individuals within families, or withholding information from the wider team in collusion with patients. Secrets play complex roles of deception and protection in families (Karpel, 1980). The intimacy of perinatal inpatient care where relationships and families are constantly assessed, observed and discussed, draws staff into the family systems of patients, with secrets or unspoken things forcing either alignment, collusion or betrayal. Within the MBU, trust and time have been required to support delineation of privacy and secrets and to reflect on the positioning of staff within the family systems of patients. Regardless, tensions continue to emerge on a day-to-day basis when juggling the needs and rights of parents and infants; including how to support the rights of parents to parent how they choose, while ensuring the rights of infants to responsive and sensitive care.

All MBU staff have had the experience of being parented, for better or worse, and many have the experience of parenting, not being able to parent, or choosing not to parent. Infants evoke strong emotions, and disagreements within the team can amplify quickly. Even with understanding of mental illness and its impacts, bearing witness to parental ambivalence or rejection, neglect or infanticidal ideation brings a different level of challenge and vicarious trauma to our team. While policies and procedures have been consistently refined and developed to support practice, in the first 12 months more time and resources than expected have been required to ensure that staff are supported. Monitoring awareness of these dynamics and tensions by unit leaders and senior staff has thus become part of the systemic work on the MBU.

**“We were juggling the baby and the bathwater”**

What seems theoretically easy in building workforce capacity, proved challenging in practice when we needed to deliver training while also delivering a high acuity clinical service. We learnt that expert positioning leads to defensiveness, as clinicians come to the MBU with their skills in other areas; not dissimilar to the patients who often come from positions of professional competence to be guzzumped by the challenges of parenthood. We have needed to develop programs and approaches to mentorship which up-skill without diminishing existing skills, and team communication approaches that support consistency while respecting different perspectives. Monthly reflective team supervision was commenced during the first year, facilitated by an experienced psychodynamic perinatal psychotherapist to enable collective space to reflect on this journey.

Alongside workforce issues, there are systemic challenges. The presence of infants and partners within the care environment has policy and governance implications for adult-centric systems. For example, we have high rates of reported ‘falls’ from infants learning to pull themselves up, despite this being a desirable and necessary milestone, yet limited ways to escalate care for partners, as they are not the primary patient. Thus, policies and procedures have required ongoing adjustments to account for relational care so that seemingly minor things (for example, a toddler stumbling) do not elicit bigger service responses than less overt but more worrying risks (for example, a parent with thoughts of harm to an infant or a coercive partner).

**“We are all left holding the baby”**

Infants require constant attention and the role of staff in providing infant care is an area of ongoing discussion. The MBU’s model of care requires that parents be ‘well enough’ to provide care of their infant 30% of the time, but this is hard to measure, assess and enforce. Infants are admitted as ‘boarders’, meaning there isn’t the funding or allocation of staff to provide direct infant care which can lead to differing understandings of the purpose of an admission amongst referrers seeking parental respite. Policies have been drafted and refined to address and communicate roles and responsibilities around infant care, however amongst the multidisciplinary team it is also not always clear whose job it is to mind infants, and in this we parallel new parent dilemmas: tensions about who is doing more, whose work is more valued, and competing needs of rest and responsibility.

## Assessment

Like all new parents, we have learnt along the way. We have learnt to make the best use of space, adjusting room layouts, sizes and usage. Alongside practical changes, we have learnt to communicate, disagree and repair ruptures in the team. We have established ongoing education and supervision, and we continue to revise policies and procedures to address all the emergent challenges. We have learnt that to provide attachment-informed care, we must also actively work to model a safe haven for staff to regulate distress, and a secure base for professional exploration.

Establishing a relational and family-oriented service within the existing paradigm of mental health services is in itself an achievement. The possibilities and challenges of doing so have long been recognised as unique to field of parental mental illness (Biebel et al., 2016). For many services, challenges relate to a lack of environmental structures, policies or dedicated staff to enact intended models (Foster & Isobel, 2018; Isobel et al., 2015). However, in the MBU, a purpose-built environment, rapidly developing policy context and enthusiastic workforce highlight other challenges to providing family-focused care within individualistic and biomedical service structures.

We believe it is important to be open about our challenges, to raise awareness of the complexity of this work, to advocate for systemic awareness of the specialist knowledge, skills, staffing and resources required to provide attachment-focused acute inpatient mental health care to parents and infants, and to ensure that we can hold the staff of the unit- so they can hold the parents admitted to the unit- so that they can hold their infants. Realistic rather than unrealistic expectations are known to be adaptive in the context of the transition to parenthood (Athan, 2024; Harwood et al., 2007) and similarly, realistic expectations may support clinicians and services as they transition to providing perinatal care within systems set up for individual adults.

## Conclusion

The analogy of the early infancy period is used to highlight the parallel process of adjustment, confidence and identity formation occurring as part of the unit development and by the parents who are admitted. Perinatal mental health care is a specialised field encompassing the health and wellbeing of parents, infants and the relational space, making it inherently more than the sum of its parts.

Opening a new unit of any kind is a challenge that requires a shuffling of the existing landscape of services and practices, as some gaps are addressed, and more gaps emerge in their wake. The establishment of a new MBU has provided an opportunity to reflect on the complexity of building workforce, service and clinical capacity within the constraints of acute public mental health services. The challenges have helped to build empathy for the experiences of our patients who equally find that things have not gone how they have planned, but that along the way there are emerging opportunities for growth, resilience and change.

## Data Availability

There is no data or material available.

## Code Aailability

There is no code available.
